# Natural sweetener *Stevia rebaudiana*: Functionalities, health benefits and potential risks

**DOI:** 10.17179/excli2021-4211

**Published:** 2021-09-22

**Authors:** Victoria Peteliuk, Lesia Rybchuk, Maria Bayliak, Kenneth B. Storey, Oleh Lushchak

**Affiliations:** 1 Department of Biochemistry and Biotechnology, Vasyl Stefanyk Precarpathian National University, 57 Shevchenko Str., Ivano-Frankivsk, 76018, Ukraine; 2 Institute of Biochemistry, Carleton University, 1125 Colonel By Drive, Ottawa, Ontario K1S 5B6, Canada; 3 Research and Development University, Shota Rustaveli Str., 76000, Ivano-Frankivsk, Ukraine

**Keywords:** Stevia rebaudiana, stevioside, rebaudioside A, sweetener, health benefits

## Abstract

*Stevia rebaudiana* is a South American plant, the cultivation of which is increasing worldwide due to its high content of sweet compounds. *Stevia* sweetness is mainly due to steviol glycosides, that are ~250-300 times sweeter than sucrose. Many studies have suggested the benefits of *Stevia* extract over sugar and artificial sweeteners, but it is still not a very popular sugar substitute. This review summarizes current data on the biological activities of *S. rebaudiana* extract and its individual glycosides, including anti-hypertensive, anti-obesity, anti-diabetic, antioxidant, anti-cancer, anti-inflammatory, and antimicrobial effects and improvement of kidney function. Possible side effects and toxicity of *Stevia* extract are also discussed.

## Abbreviations

CFTR, cystic fibrosis transmembrane conductance regulator; GFR, glomerular filtration rate; LPS, lipopolysaccharide; MDCK cells, Madina-Darby canine kidney cells; NF-kB, nuclear factor kappa-light-chain-enhancer of activated B cells; Nrf2, nuclear erythroid factor 2; TRPM5, transient receptor potential cation channel subfamily melastatin member 5; TPA, 12-O-tetradecanoyl-phorbol-13-acetate.

## Introduction

The genus *Stevia* of the *Asteraceae* family includes 230 species, but only one of them, *Stevia rebaudiana* Bertoni produces sweet steviol glycosides. It was earlier thought that *S. phlebophylla* also has this property, but new research denies this (Brandle and Telmer, 2007[17]; Ceunen et al., 2013[21]).

*S. rebaudiana *is a perennial shrub growing in South America, in particular in Brazil and Paraguay, where it is also known as “Honey Leaf”, “Sweet-Leaf” or “Sweet-Herb”. *Stevia* preparations are used in many forms such as fresh and dried *Stevia* leaves, *Stevia* leaf powder, extracts and liquid concentrates. *Stevia* extract is a great alternative for synthetic sweeteners being approximately 200 to 300 times sweeter than sugar (Singh and Rao, 2005[100]). Many studies have shown that *Stevia* leaf preparations as a natural non-calorie sugar substitute is not only safe for people with diabetes, high blood pressure, and obesity but also can be used for the treatment of these diseases or prevention of their complications (Carrera-Lanestosa et al., 2017[19]). Safety of the consumption of the herb by children was also shown (Aguero et al., 2014[4]). 

**Figure 1:**
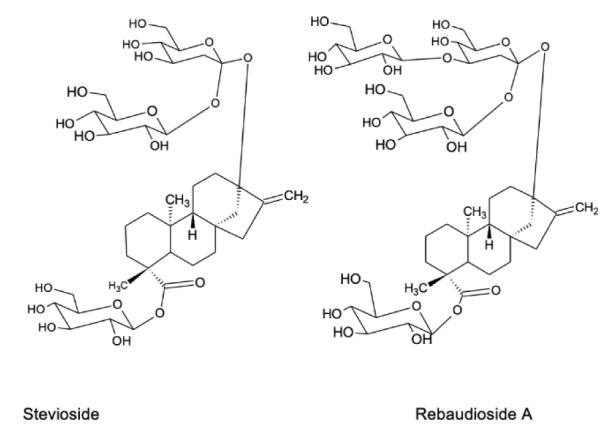
Chemical structure of two main compounds of *Stevia* leaves responsible for their sweet taste

The main compounds responsible for the sweetness of *Stevia* preparations are steviol diterpene glycosides (Figure 1(Fig. 1)) called stevioside and rebaudiosides, and they are used in the food industry as sweeteners. In addition to sweet compounds, *Stevia* leaves contain many other biologically active substances, which have beneficial effects for human health. In particular, the anti-diabetic, antihypertensive, antitumor, anti-cariogenic, anti-inflammatory and bactericidal effects of the herb have been studied, and these issues are discussed in detail below. There are also data on the protective effects of *Stevia* on the digestive system and skin disorders as well as on common complications associated with metabolic syndrome (Singh and Rao, 2005[100]; Sanches Lopes et al., 2016[93]; Ranjbar et al., 2020[86]).

## History of Stevia in the World

*Stevia* has been known to people from ancient times. The Guarani Indians called *Stevia* “Ka-a He-e”, which means “sweet grass”, and used it to savor bitter drinks such as mate. There are some reports that *Stevia* was already known in Spain in the 16^th^ century. However, other Europeans learned about the plant only in the late 19^th^ century, after *Stevia* was introduced and promoted by the botanist Moises Santiago Bertoni (Ramesh et al., 2006[85]). Bertoni renamed it from *Eupatorium rebaudianum* to *Stevia rebaudiana *(Carakostas et al., 2008[18]). In 1901, Bertoni wrote that a few leaves of *Stevia* grass were enough to sweeten a large cup of tea. In 1920, *Stevia* began to be cultivated in large quantities on plantations in Brazil and Paraguay. In 1931, the French chemists Briedel and Lavieille isolated the glycoside which provide *Stevia* with its sweet taste. This compound was named stevioside (Barriocanal et al., 2008[13]). During World War II, *Stevia* began to be actively consumed in the United Kingdom due to sugar shortages and rationing of its consumption (Zaman et al., 2015[119]).

In the 1970s, *Stevia* was introduced in Japan and research was started to evaluate its beneficial potential for human health. Since then, the Japanese actively use this sweetener in a variety of foods and Japan is one of the major producers of *Stevia* now (Ramesh et al., 2006[85]). In 2013, the Coca-Cola Company started production of drinks containing* Stevia* instead of sugar and having 30 % less calories. These drinks are now sold in a number of countries worldwide (Heyden, 2013[41]). In addition to Japan, Brazil, and Paraguay, *Stevia* is commercially cultivated in many other countries around the world, including Ukraine (Midmore and Rank, 2002[64]). 

## Biologically Active Compounds of Stevia

The leaves of *S. rebaudiana* contain more than 30 different steviol glycosides, among which stevioside and rebaudioside A are present in the highest levels (Figure 1(Fig. 1)). Stevioside and rebaudioside A are also the main sweet components of *Stevia*. Stevioside was first isolated from *Stevia* in 1931 and its chemical structure was established in 1952. Stevioside is a diterpene glycoside consisting of three molecules of glucose and a glucone moiety - steviol. Stevioside content is from 4 % to 13 % of all glycosides in *Stevia *(Marcinek and Krejpcio, 2015[56]). Rebaudioside A is another steviol diterpene glycoside; the percentage of its sweetness fluctuates from 30 % to 40% that is ~ 180-400 times sweeter than sugar (Kaplan and Turgut, 2019[47]). The concentration of stevioside is 6.5 - 9.1 % and rebaudioside A is 2.3 - 3.8 % (Atteh et al., 2011[12]; Goyal et al., 2010[39]).

In total, the best-known compounds that have been isolated from *Stevia *leaves are glycosides, stevioside, several types of rebaudiosides (from A to F), steviolmonoside, rubusoside, dulcoside A, and steviolbioside (Starratt et al., 2002[102]; Savita et al., 2004[95]). Less common are penta-glucoside rebaudioside D and hexa-glucoside rebaudioside M (Olsson et al., 2016[71]). Rebaudioside D and rebaudioside M are present in dried *S. rebaudiana* leaves in very low quantities of 0.2 % and 0.1 %, respectively (Neuwirth, 2020[68]). Despite that, rebaudioside M is commercialized by PureCircle Limited and the Coca-Cola Company for food and beverage use (Prakash et al., 2014[81]). Rebaudioside D can also be used in the food industry as a non-caloric sweetener (Allen et al., 2013[8]).

Recently, other rebaudiosides have been identified in *Stevia*, namely rebaudioside U, T, R and S (Ibrahim et al., 2016[43]; Perera et al., 2017[76]). Steviol and isosteviol, which are products of hydrolysis of stevioside can be used for therapeutic purposes (Wang et al., 2018[116]). Most of these isolated diterpenoid glycosides have the same chemical backbone structure (steviol) but differ in the residues of carbohydrate at positions C13 and C19 (Shibata et al., 1995[97]; Purkayastha and Kwok, 2020[84]). Sweet *Stevia* glycosides differ both in the degree of sweetness and in quantitative content in the leaves. The most abundant glycosides, stevioside and rebaudiosides, are the sweetest compounds of *Stevia* and in the refined form are in 150-300 and 250-450 times sweeter than sucrose, respectively (Marcinek and Krejpcio, 2015[56]). Processed forms of *Stevia* leaves are generally 250-300 times sweeter than sugar. 

In addition to sweet compounds, *Stevia* leaves contain proteins, carbohydrates, lipids, dietary fibers, oils, vitamins, and phenolic compounds. Dried *Stevia* leaves contain (per 100 g of dried mass) 11.2-16.0 g proteins, 61.9 g carbohydrates, 1.9-3.73 g lipids and 6.8-15.2 g dietary fiber (Abou-Arab et al., 2010[2]; Goyal et al., 2010[39]; Atteh et al., 2011[12]).

The chemical composition of preparations from *Stevia* differs in dry and fresh leaves and depends on the method of processing or extraction (Snehal and Madhukar, 2012[101]). 

In addition, the biochemical composition of the plant depends on the geographical region of the growth (Table 1(Tab. 1)) (Khiraoui et al., 2017[48]). It was found that *Stevia* leaves contain a number of phenolic compounds that exhibit strong antioxidant properties. The content of total polyphenols and flavonoids in methanolic extracts was estimated as 25.18 mg/g and 21.73 mg/g, respectively (Tadhani et al., 2007[103]). This herb contains also oils, which are rich in palmitic, palmitoleic, stearic, oleic, linoleic and linolenic fatty acids (Table 2(Tab. 2)). *Stevia* leaves are also an important source of water-soluble vitamins including vitamin C (14.98 mg/100 g), vitamin B2 (0.43 mg/100 g), folic acid (52.18 mg/100 g) (Kim et al., 2011[49]). Moreover, the plant is rich in macro- and microelements such as Zn, Fe, Ca, K, Na, Mg and other minerals that are essential for human health (Table 3(Tab. 3)).

It should be noted, that not only *Stevia* contains stevial glycosides. *Rubus suavissimus* also known as sweet tea, contains steviol monoside, rebaudioside A, B, C, D, F, M, stevioside, steviolbioside, and rubusoside (Uhler and Yang, 2018[108]). In addition, *R. suavissimus* is rich in its own glycosides called suaviosides B, C, D1, D2, E, F, G, H, I and J (Ohtani et al., 1992[70]). 

## Health Benefits of Stevia

**Figure 2:**
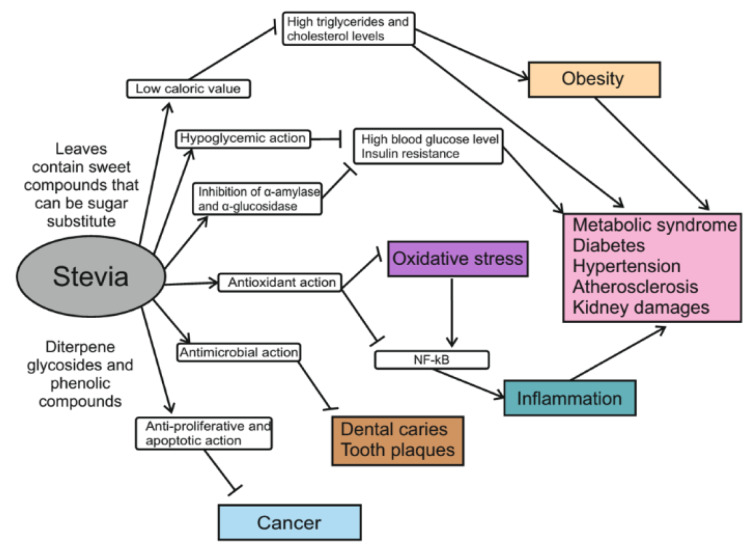
Schematic summarizing of health-promoting effects of *Stevia* leaf extract and its constituents

The properties of *Stevia* have been studied for over 100 years. Both earlier and current studies not only confirm the safety of *Stevia *leaf preparations but also find more and more benefits of its consumption for human health. Next, we will summarize current data on the established and potential pharmacological effects of *Stevia* extracts and its individual components in various medical models (Table 4(Tab. 4); References in Table 4: Abo Elnaga et al., 2016[1]; Abudula et al., 2004[3]; Ahmad and Ahmad, 2018[5]; Boonkaewwan et al., 2008[15]; Casas-Grajales et al., 2019[20]; Chan et al., 1998[22]; Chen et al., 2018[24]; Dyrskog et al., 2005[29]; Ferri et al., 2006[32]; Geeraert et al., 2010[36]; Ghanta et al., 2007[37]; He et al., 2019[40]; Hsieh et al., 2003[42]; Lailerd et al., 2004[52]; Maki et al., 2008[55]; Mehta et al., 2011[58]; Melis, 1992[62], 1995[59], 1997[61]; Misra et al., 2011[66]; Park and Cha, 2010[74]; Philippaert et al., 2017[79]; Prata et al., 2017[82]; Ranjbar et al., 2020[86]; Rizwan et al., 2018[88]; Rotimi et al., 2018[89]; Roy et al., 2010[90]; Ruiz-Ruiz et al., 2015[92][91]; Sánchez-Aceves et al., 2017[94]; Shukla et al., 2012[98]; Singh and Dwivedi, 2013[99]; Takasaki et al., 2009[105]; Yuajit et al., 2013[118], Figure 2(Fig. 2)).

### Antioxidant properties of S. rebaudiana

Free radicals, which are continuously formed in any organism as result of metabolic processes or exposure of various stresses, may contribute a large number of human diseases, including cancer, obesity, diabetes, and neurodegenerative diseases (Garaschuk et al., 2018[34]; Bayliak et al., 2019[14]; Vaiserman et al., 2020[110][111]). Under physiological conditions, the capacity of endogenous antioxidant defense is sufficient to neutralize free radicals and prevent oxidative damages, but aging and excessive caloric intake are accompanied by imbalance between the production and elimination of free radicals followed by chronic oxidative stress and systemic inflammation development (Garaschuk et al., 2018[34]; Bayliak et al., 2019[14]; Vaiserman et al., 2020[111]). Therefore, using bioactive phytochemicals having antioxidant and anti-inflammatory activities is considered as a promising therapeutic approach to combat aging and associated pathological conditions (Michels et al., 2018[63]; Piskovatska et al., 2019[80]; Vaiserman et al., 2020[110][111]). Many studies showed that the antioxidant properties of *S. rebaudiana *might determine its ability to prevent and treat these diseases. *Stevia* leaves contain a number of phenolic compounds that are able to neutralize free radicals and chelate transition metal ions, thus preventing the involvement of the latter in free radical generation via the Fenton reaction (Ruiz*-*Ruiz et al., 2015[92]; Prata et al., 2017[82]).

Viability of rat cardiac fibroblasts pre-treated with glycosides of steviol was significantly increased under H_2_O_2_ exposure compared with the control cell group (Prata et al., 2017[82]). These cells also showed an increase in levels of reduced glutathione, activities of superoxide dismutase 1 and catalase (Prata et al., 2017[82]). One study found that *Stevia *aqueous leaf extract was able to reduce the levels of 1,1-diphenyl-2-picrylhydrazyl-hydroxyl radical, nitric oxide and superoxide anion radicals* in vitro*, although the effect was slightly lower compared to the antioxidant potential of ascorbic acid that was used as a positive control (Shukla et al., 2012[98]). Another study showed that ethyl acetate extract and crude 85 % methanolic extract from *S. rebaudiana* leaves prevented the formation of DNA breaks caused by free radicals (Ghanta et al., 2007[37]).

*Stevia* glycosides were found to reduce lipoperoxidation and decrease hydroperoxide and protein carbonyl content in *Cyprinus carpio* (Sánchez-Aceves et al., 2017[94]). Stevioside significantly reduced oxidative stress by decreasing the levels of lipid peroxidation and nitric oxide in the liver and kidney of diabetic rats (Rotimi et al., 2018[89]). In addition, stevioside has been shown to inhibit thioacetamide toxicity in rat liver (Casas-Grajales et al., 2019[20]). Prolonged administration of thioacetamide to rats caused damage to the liver and altered the content of nuclear erythroid factor 2 (Nrf2) that led to a decrease in antioxidant capacity of the liver. Stevioside prevented thioacetamide-induced liver damage by regulation of the level of Nrf2 protein (Casas-Grajales et al., 2019[20]). 

The antioxidant properties of *Stevia* extracts depend on the methods of extract processing including stages of drying and extraction. Thus, the highest total content of phenols was observed in glycol-aqueous extracts from *Stevia* leaves, with lower phenol content in aqueous extracts and the lowest levels in ethanol extracts (Gaweł-Bęben et al., 2015[35]). Comparison of the antioxidant capacity of *Stevia* leaves dried by different methods (hot air drying at 100 °C, hot air drying at 180 °C, freeze drying, shade drying) showed that the best method of drying to keep high antioxidant potential was hot air drying at 180 °C. Under this regime, the content of antioxidants was 2-3 times higher than when using other drying methods (Periche et al., 2015[77]).

### Anti-cancer effects 

Cancer is the second leading cause of mortality in the world. The most common forms of cancers in 2017 were breast cancer, colorectal cancer, prostate cancer, and lung cancer. Therefore, the search for agents that may help in cancer treatment or prevention is extremely important (Ferlay et al., 2018[31]).

**Figure 3:**
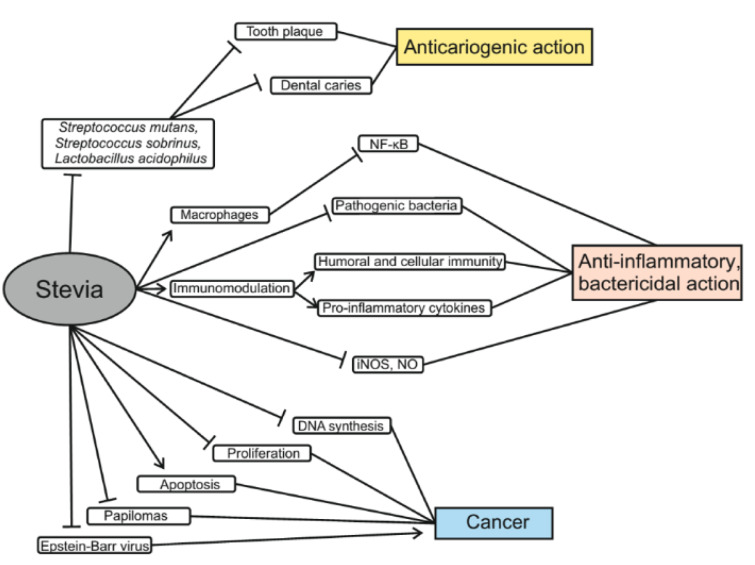
Antimicrobial, anti-inflammatory and anti-cancer effects of *Stevia* extracts

The results of one experiment showed that steviol inhibited the proliferation of six types of human cancer cells of the gastrointestinal tract (Chen et al., 2018[24]). High concentrations of stevioside were shown to reduce the viability of colon cancer cells (Boonkaewwan et al., 2008[15]). At a concentration of 100-200 μg/mL steviol acts with similar efficacy as 5-flourouracil (anti-cancer drug), and at a concentration of 250 μg/mL, it showed even greater cytotoxicity than 5-FU. Accordingly, steviol may become a potential chemotherapeutic agent for cancer treatment (Chen et al., 2018[24]). It is also worth noting that stevioside is less toxic to normal cells even at higher doses. Another study showed that stevioside could inhibit DNA synthesis and induce death of cancer cells via the mitochondrial apoptotic pathway. This is because steviol increases the expression of p21 and p53 proteins and decreases Cyclin D. As a result, it leads to up-regulation of the Bax/Bcl-2 ratio. Bax protein, in turn, leads to apoptosis associated with dysfunction of mitochondria and release of cytochrome c, which causes the activation of caspases that then cleave enzymes involved in repairing DNA and maintaining genome integrity (Paul et al., 2012[75]; Chen et al., 2018[24]). Due to this, stevioside showed a strong anti-cancer activity in cultured breast cancer MCF-7 cells (Paul et al., 2012[75]). In mice, combined treatment with stevioside and carcinogens, 7,12-dimethylbenzanthracene and 12-O-tetradecanoyl-phorbol-13-acetate (TPA), for 20 weeks, reduced papilloma formation by 94 % (Konoshima and Takasaki, 2002[50]). Similar results were obtained in another study that reported a three-fold reduction in the formation of tumors on skin cell meshes exposed to stevioside as compared with mice treated with 7,12-dimethylbenzanthracene plus TPA only (Yasukawa et al., 2002[117]). In addition, steviol, isosteviol and their metabolites block the induction of an early Epstein Barr virus antigen that inhibits tumor progression (Takasaki et al., 2009[105]). Thus, all of these studies support that effectiveness of steviol and other *Stevia* components in chemotherapy of cancer (Mizushina et al., 2005[67]) (Figure 3(Fig. 3)). 

### Anti-inflammatory and bactericidal action of Stevia. Anti-cariogenic properties

Recent studies demonstrate that* S. rebaudiana *is a unique plant with anti-inflammatory and bactericidal properties (Figure 3(Fig. 3)). *Stevia* extract inhibits the activity of many pathogenic bacteria, can be used to treat immune diseases and reduce edema (Preethi et al., 2011[83]; Jeong et al., 2010[45]; Arya et al., 2012[10]).

It was found that treatment of RAW 264.7 macrophage cells with the ethyl acetate fraction from *Stevia *leaves significantly inhibited the NF-κB-mediated gene expression that was stimulated by bacterial lipopolysaccharide (LPS). Such inhibition of NF-κB signaling was closely associated with a decrease in levels of interleukin-6 and monocyte chemoattractant protein-1. It was suggested that *Stevia* could be used for treatment of immune diseases such as rheumatoid arthritis and lupus (Jeong et al., 2010[45]). Stevioside was found to exhibit immunomodulatory effects and can act by stimulation of both humoral and cellular immunity and phagocytic function (Sehar et al., 2008[96]). Stevioside and steviol attenuate LPS-induced pro-inflammatory cytokine production by affecting cytokine gene expression via the IκBα/NF-κB signaling pathway (Boonkaewwan and Burodom, 2013[16]). Austroinulin and 6-O-acetyl-austroinulin are other natural diterpenoids isolated from *S. rebaudiana* with anti-inflammatory activity. It was found that these diterpenoids inhibit NO production and iNOS expression by blocking the activation of STAT1, IRF3, and NF-κB in LPS-stimulated RAW264.7 macrophages (Cho et al., 2013[25]). The methanolic extract of callus culture as well as the intact plant part of *Stevia *significantly inhibited carrageenan-induced rat paw edema (Preethi et al., 2011[83]; Arya et al., 2012[10]) analyzed the antibacterial activity of *Stevia* extracts *in vitro* against pathogenic bacteria such as *Bacillus subtilis*, *Klebsiella pneumonia*, *Proteus vulgaris*, *Streptococcus pneumoniae*, *Staphylococcus aureus* and *Pseudomonas fluorescence*. Six extracts from the leaves and three extracts from* Stevia* flowers obtained using of different solvents showed good antibacterial activity against all tested microorganisms.

*Stevia* also has antibacterial effects in the oral cavity (Gamboa and Chaves, 2012[33]). Dental caries is a common disease that affects not only adults but also children. Tooth caries occur due to poor oral hygiene, hereditary predisposition, consumption of foods rich in carbohydrates, or low levels of calcium and phosphorus in the body. Poor oral hygiene and consumption of carbohydrates promotes active reproduction of mouth microbiota following microbial colonization and biofilm and plaque formation on teeth. *In vitro* studies have shown that *S. rebaudiana *stops the growth of *Streptococcus mutans, S. sobrinus *and* Lactobacillus acidophilus*. These bacteria are associated with the development of caries (Contreras, 2013[26]).

Gamboa and Chaves (2012[33]) also studied the activity of different *Stevia* extracts against microorganisms that cause tooth decay. Extracts were obtained from dried *S. rebaudiana *leaves using hexane, methanol, ethanol, ethyl acetate or chloroform as solvents. The antimicrobial activity of these five extracts against 16 bacterial strains of the genera *Streptococcus* and *Lactobacillus* was evaluated by the well-known diffusion method. For the four *Lactobacillus* species, the inhibition zones obtained between 12.3 and 17.3 mm were somewhat larger with ethyl acetate and chloroform extracts, suggesting that these bacteria were the most susceptible microorganisms. In another study, four groups of rats were fed with stevioside, rebaudioside A or sucrose, which were added to the main diet. There were significant differences in the rates of sulcal caries and *Streptococcus sobrinus* count between a group fed diet with 30 % sucrose and the other 3 groups. There were no differences between groups fed stevioside or rebaudioside A and groups without additives. Thus, it can be argued that neither stevioside nor rebaudioside A are cariogenic (Das et al., 1992[27]). An *in vivo* study in humans was performed by measuring the plaque formation after rinsing with sucrose solution and *S. rebaudiana* solution four times a day for 5 days. The plaque accumulation after rinsing with *Stevia* solution was 57.82 % less than when rinsing with sucrose, measured by the Silness-Loe index, and 10.40 % less plaques when measured via the O'Leary plaque index (De Slavutzky, 2010[28]). Caries-positive and caries-negative controls were used for another experiment. That experiment found, that *Stevia* extract solution reduced the number of viable microbial cells compared to sucrose, suggesting that *Stevia* prevents growth of bacteria in the mouth (Giacaman et al., 2013[38]). Due to observed antimicrobial activity, *Stevia* was proposed for use to prevent dental caries (Ма and Blanksma, 2015[54]). Moreover, *Stevia *was shown to have strong anti-plaque and anti-gingivitis properties (Vandana et al., 2017[115]).

## Stevia in the Treatment of Diabetes

Diabetes is a group of diseases in which insulin is either not produced in sufficient quantities (type 1 diabetes) or cannot be used effectively due to insulin resistance (type 2 diabetes). As of 2019, there were 464 million people with diabetes in the world; for comparison, in 2000, 175.4 million of patients with diabetes was recorded. According to the International Diabetes Federation, by 2040 this number will increase to 642 million (Zimmet et al., 2016[120]). About 90 % of patients are diagnosed with type 2 diabetes in both developing and developed countries (Tao et al., 2015[106]; Vaiserman and Lushchak, 2019[112]). Major risk factors contributing to the development of type 2 diabetes include genetic predisposition, unhealthy dietary behaviors, and low physical activity. The pathophysiology of type 2 diabetes is characterized by impaired glucose metabolism in the liver and insulin resistance in peripheral tissues which show reduced responsiveness to normal insulin concentrations and, therefore, cannot utilize glucose. As a result, glucose levels are increased in the blood in patients with type 2 diabetes, despite increased insulin production by the pancreas (Vaiserman and Lushchak, 2019[112][113]).

**Figure 4:**
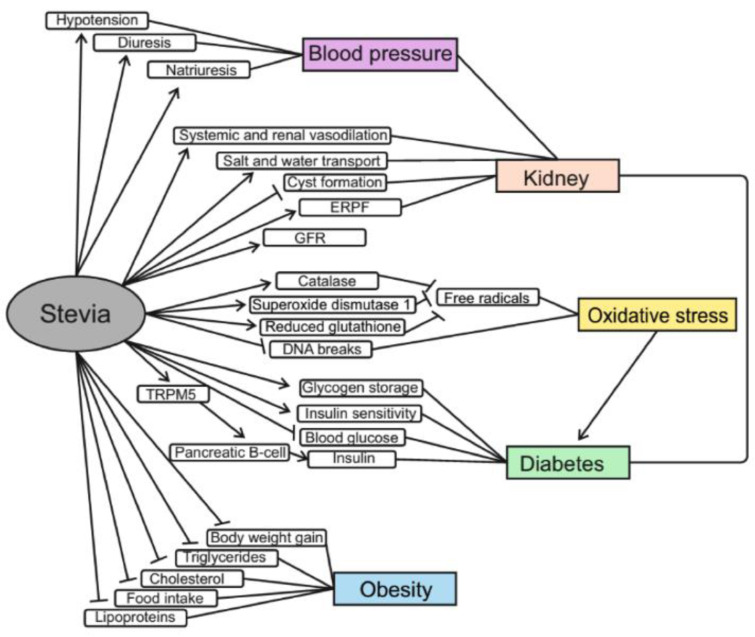
Protective effects of *Stevia* extracts in obesity, diabetes and its complications

For many years, *Stevia* has been used not only as a sweetener but also as a medication in the treatment of diabetes and hyperglycemia in traditional medicine in Brazil and Paraguay (Chand and Kumar, 2016[23]). The anti-diabetic properties of *Stevia* leaf extracts and its individual ingredients are now being actively investigated (Figure 4(Fig. 4)). One study showed that diabetic rats that consumed an aqueous extract from *Stevia* leaves had higher levels of insulin and glycogen than animals of the control group (Ahmad and Ahmad, 2018[5]). An increase in insulin production was also observed in isolated mouse islets of Langerhans treated with rebaudioside A (Abudula et al., 2004[3]). In addition, stevioside can increase insulin sensitivity; in particular, low amounts of stevioside improved the effect of insulin on glucose transport into skeletal muscle (Lailerd et al., 2004[52]). Glycosylated hemoglobin in experimental groups of rats was significantly decreased as compared with diabetic control groups and was near to the value for non-diabetic rats (Lailerd et al., 2004[52]). Consumption of *Stevia* aqueous extract for 8 weeks resulted in a significant decrease in random blood glucose by 73.2 % and fasting blood glucose by 66.1 % (Ahmad and Ahmad, 2018[5]). A number of studies have also shown significant reduction in blood glucose levels after consumption of *Stevia* extract (Roy et al., 2010[90]; Mehta et al., 2011[58]; Singh and Dwivedi, 2013[99]; He et al., 2019[40]). *Stevia* extract has been found to improve pancreatic β-cell function in diabetic rats due to the ability of steviol glycosides to increase glucose-induced Ca^2 +^ oscillations and insulin release by pancreatic islets (Misra et al., 2011[66]) This mechanism is based on the interaction of steviols, rebaudioside A and stevioside with the transient receptor potential cation channel subfamily melastatin member 5 (TRPM5). These receptors play an important role in the transmission of bitter, sweet and umami flavors and are localized on type II taste receptor cells and pancreatic β-cells. TRPM5 channels are activated by increasing the content of intracellular calcium and increased ATP release and afferent signaling from the type II taste receptor cell. In the case of β-cells, increasing the activity of TRPM5 leads to an increase in the frequency of oscillations of *V*_m_ and [Ca^2+^]_cyt_, which promotes insulin secretion (Philippaert et al., 2017[79]). Moreover, rats with hyperglycemia, after administration of *Stevia *aqueous extract, consumed significantly less water compared to other hyperglycemic rats (Ahmad et al., 2018[6]). Other studies have shown that stevioside and steviol may have potential in the treatment of diabetes because they act as anti-hyperglycemic agents by directly affecting β-cells of the pancreas and their insulinotropic action stopped when blood glucose level is dropped to the normal level (Jeppesen et al., 2000[46]). The absence of insulin-stimulating effects at normal glucose levels may reduce the risk of hypoglycemia (Jeppesen et al., 2000[46]). Groups of diabetic mice that consumed the antioxidant fraction of *Stevia *supplements showed better glucose tolerance than the control group (Milani et al., 2017[65]). Furthermore, *Stevia* leaf extract was found to be able to inhibit α-amylase and α-glucosidase. This can potentially slow down carbohydrate metabolism and reduce the risk of hyperglycemia in patients with type 1 and type 2 diabetes (Ruiz-Ruiz et al., 2015[91]). However, some studies on humans found no effects of supplements of* Stevia* aqueous extract on glucose, insulin, and glycosylated hemoglobin levels, but also no side effects (Barriocanal et al., 2008[13]; Ajami et al., 2020[7]).

### Anti-hypertension effects of Stevia

High blood pressure or hypertension is the most dangerous factor in the development of myocardial infarction and ischemic stroke. It can be a separate disease or a symptom of chronic diseases of kidney and endocrine organs, some heart defects, atherosclerosis, organic lesions of the central nervous system, *etc*. (Oparil et al., 2018[73]). 

In one study, the effect of intravenous stevioside on blood pressure was studied in spontaneously hypertensive rats (SHR). The hypotensive effect on both systolic and diastolic blood pressure was dose-dependent for intravenous doses of 50, 100 and 200 mg/kg in conscious SHR. The hypotensive effect lasted for more than 60 min with a dose of 200 mg/kg. Thus, stevioside was effective in blood pressure reduction (Chan et al., 1998[22]). In another study, *S. rebaudiana* extracts were administered to rats for 20, 40, and 60 days and results showed that rats treated with *Stevia *extract for 20 days did not significantly differ from the control group in the arterial pressure (Melis, 1995[59]). However, after 40 or 60 days of the administration, the extract caused hypotension, diuresis and natriuresis with a constant glomerular filtration rate. Steviol treatment did not cause significant changes in mean blood pressure in rats, diuresis and natriuresis with a constant glomerular filtration rate (Melis, 1997[61]). In one study, patients took capsules containing 500 mg stevioside powder or placebo three times daily for 2 years. This study shown that in patients with mild hypertension, oral stevioside significantly decreased systolic blood pressure and diastolic blood pressure compared with placebo (Hsieh et al., 2003[42]). Consumption of 1000 mg/day of rebaudioside A caused no clinically important changes in blood pressure in healthy adults with normal and low-normal blood pressure (Maki et al., 2008[55]). Rebaudioside A did not affect blood pressure or glycemic control when Goto-Kakizaki rats consumed this glycoside (0.025 g/kg body mass/day) for eight weeks (Dyrskog et al., 2005[29]). Systolic and diastolic blood pressure decreased in humans during treatment with crude stevioside, but a similar effect was observed in the placebo group (Ferri et al., 2006[32]).

### Stevia effects on obesity 

At present, obesity is considered to be a serious global problem, and the number of obese and overweight people continues to increase worldwide. However, obesity is not just overweight, but it is a complex disease with many metabolic complications, such as diabetes, cancer, cardiovascular diseases, stroke, and sleep apnea. Obesity significantly increases the risk of these diseases leading eventually to impaired quality of human life (Bayliak et al., 2019[14]). Abdominal obesity is one of the factors of metabolic syndrome, that increases a risk of cardiovascular diseases (Carrera-Lanestosa et al., 2017[19]; Bayliak et al., 2019[14]). These factors include hepatic steatosis and dyslipidemia. Research shows that consumption of non-nutritive sweeteners can prevent body weight gaining by reducing energy intake (Ashwell, 2015[11]). In line with this, studies with *S. rebaudiana *support its anti-obesity properties (Figure 4(Fig. 4)). In one 12-week experiment on rats, it was found that oral administration of *Stevia* sweetener at doses of 25, 250, 500 and 1000 mg/kg body mass decreased body weight gain by 40.3, 41.4, 45.0 and 48.3 %, respectively (Abo Elnaga et al., 2016[1]). This may be due to the reduced food intake after administration of *Stevia* sweetener. *Stevia *sweetener intake also reduced total cholesterol, triglycerides and low-density lipoprotein concentrations and increased high-density lipoprotein levels in the blood of experimental rats (Abo Elnaga et al., 2016[1]). *S. rebaudiana* extract supplementation decreased serum and liver triacylglyceride le-vels in mice fed a high-fat diet compared to mice fed the high fat diet without adding *Stevia* extract (Park and Cha, 2010[74]). In addition, mice kept on a high-fat diet with *Stevia* extract showed an increase in carnitine levels, which plays an important role in fatty acid metabolism as it transports fatty acids from cytosol to mitochondria where they are oxidized (Park and Cha, 2010[74]). 

Another human study found that the significant difference in calorie intake between the control group and the group that consumed *Stevia* extract was only due to the difference in calorie intake; therefore, consumption of *Stevia* extract did not affect the feeling of hunger and appetite (Farhat et al., 2019[30]). It also can explain why the participants in another human experiment did not consume additional food to compensate for the lack of calories under *Stevia *extract administration in contrast to participants who consumed sucrose (Anton et al., 2010[9]). 

Obesity is frequently associated with insulin resistance and increased inflammation and oxidative stress. One study showed that stevioside treatment was associated with inhibition of atherosclerotic plaque development via improvement of insulin signaling and antioxidant defense in obese insulin-resistant mice (Geeraert et al., 2010[36]). In addition, it was shown that *Stevia* extract could prevent the adverse effects of high fat, high sucrose diet on lipid profiles, total antioxidant capacity and histopathologic factors in obese rats (Ranjbar et al., 2020[86]).

### Renal function 

The kidneys are one of the most important organs, their main function being to excrete end- and side-products of metabolism. The kidneys are also involved in regulation of blood pressure, water-salt balance and red blood cell formation (Onopiuk et al., 2015[72]). Diabetes is the leading cause of kidney disease. In turn, diabetic kidney disease (DKD) is the leading cause of kidney failure worldwide. People with diabetes most often die from renal failure (Reidy et al., 2014[87]). High glucose concentrations induce specific cellular changes, which affect many types of cells in the kidney, including endothelial cells, smooth muscle cells, mesangial cells, podocytes, cells of the tubular and collecting duct system, and inflammatory cells and myofibroblasts (Vallon and Komers, 2011[114]). 

Experiments with normal and hypertensive rats showed that stevioside caused hypotension, diuresis and natriuresis in both groups (Melis, 1992[62]). The first (control) group demonstrated an increase in renal plasma flow and glomerular filtration rate constant following stevioside administration. For the group that consumed the extract for 60 days, an increase in renal plasma flow was observed. In addition, it was shown that the aqueous extract from dried *Stevia* leaves caused systemic and renal vasodilation, hypotension, diuresis and natriuresis (Melis, 1995[59]). Yuajit et al. (2013[118]) investigated inhibitory effects and the detailed mechanisms of action of steviol and its derivatives on cyst growth using a cyst model, the Madina-Darby canine kidney (MDCK) cell line. Taken together, the data suggest that steviol delays the progression of MDCK cysts by directly inhibiting cystic fibrosis transmembrane conductance regulator (CFTR) chloride channel activity and reducing CFTR expression, in particular by promoting proteasomal degradation of CFTR. Therefore, steviol and related compounds can be further used to treat polycystic kidney disease.* Stevia *extract improves some biochemical parameters of patients with chronic kidney disease; in particular, patients have improved serum creatinine, uric acid, and blood sugar before meals (Rizwan et al., 2018[88]). Steviol elicited no significant changes in glomerular filtration rate (GFR) and renal effective plasma flow (ERPF). However, the steviol infusion induced a significant increase in fractional sodium excretion, fractional potassium excretion, and urinary flow as a percentage of glomerular filtration rate (V/GFR) when compared to controls. The data suggest that steviol may affect salt and water transport in renal tubules (Melis, 1997[61]).

## Toxicity and Side Effects

The acceptable daily intake of *Stevia* dry extract defined by the Scientific Committee on Food of the European Food Safety Authority and Food and Drug Administration is 4 mg/kg body mass (Lohner et al., 2017[53]). One animal study showed the allergenic potential of *Stevia *preparations. It is possible that crude *Stevia* extracts have a higher allergenic potential than high purity stevia-based sweeteners containing ≥ 95 % steviol glycosides, since crude extracts are more likely to contain allergenic substances inherent in the *Asteraceae* family (Urban et al., 2015[109]), but this issue is not well studied yet. 

According to the results of one earlier study, *Stevia* extract reduced the fertility of rats by up to 21 % compared with control group of rats. Fertility remained reduced by 47 % even after a 50-60 day recovery period (Mazzei-Planas and Kuc, 1968[57]). In *Stevia*-treated rats, a decrease in the relative weight of seminal vesicle and testis was observed as well as a significant decrease in the number of spermatozoa stored (Melis, 1999[60]). 

One study found that steviol is mutagenic, although other studies have not confirmed this effect (Pezzuto et al., 1985[78]). The main evidence of *Stevia* safety is that for over 1500 years of continuous use by Paraguayans, there have been any reports of adverse effects. Additional confirmation of the safety of *Stevia* consumption is the absence of reports on side effects of any kind in Japanese populations where *Stevia* has been consumed in large quantities in recent years (Singh and Rao, 2005[100]). In addition, most of the studies that have investigated *Stevia* effects on the human body have showed no side effects (Roy et al., 2010[90]; Nikiforov et al., 2013[69]; Uçar et al., 2017[107]). However, it should be noted that not all of the *Stevia* products sold are of high quality. In particular, in the one experiment Raman spectra of six commercial products of *Stevia* were measured and it was found that three of the commercial *Stevia *products were counterfeit products. They also contained sodium cyclamate and small amounts of sodium saccharin (Jentzsch et al., 2016[44]).

## Conclusions

*S. rebaudiana*, a perennial plant native to Paraguay, has come to be cultivated around the world as a source of high-potency sweetener with no caloric value*. *Two main steviol diterpene glycosides, stevioside and rebaudioside A, that are present in high levels in Stevia leaves provide the sweet taste of the plant and are 150-450 times sweeter than sucrose to human taste buds. Pure sweet glycosides and crude *Stevia* extracts with 50 % of glycosides are actively used in the food market. A number of preclinical and clinical studies suggest potential therapeutic and pharmacological applications for *Stevia* extracts and their individual compounds because they demonstrate no toxicity in experimental trails and exhibit health-promoting activities. In addition to different glycosides, *Stevia* leaves contain many other compounds like flavonoids and fatty acids that together provide the diverse biological properties of the plant. Thanks to these components,* Stevia* products stimulate insulin production in diabetics, improve polycystic kidney disease, have chemotherapeutic action in cancer and possess powerful antibacterial, antioxidant and immunomodulating properties (Figure 2(Fig. 2)). More research is needed to elucidate which compounds are the main determinants of the known *Stevia*-based effects as well as their molecular mechanisms of action. In addition, mechanisms need to be established by which *Stevia* sweetener reduces food intake and lowers total cholesterol, triacylglycerides, and low-density lipoproteins.

## Notes

Victoria Peteliuk and Lesia Rybchuk contributed equally as first authors.

## Ethical statement

This is a review paper, which does not include animal or human experiments. 

## Funding

This work was partially supported by the Ministry of Education and Science of Ukraine (grants #0117U006426 and #0118U003477) and a Discovery grant from the Natural Sciences and Engineering Research Council of Canada (#6793).

## Conflict of interest

The authors declare that they have no conflict of interest.

## Authors’ contributions

**Victoria Peteliuk** and **Lesia Rybchuk** collected literature and wrote the original draft, prepared the figures and tables; **Maria Bayliak** performed analysis, review and edited the manuscript, helped to prepare and arrange tables and figures; **Kenneth Storey** performed review, editing and provided valuable discussion; **Oleh Lushchak**: provided funding acquisition, idea and design of the article, supervising, review, and editing. All authors read and approved the final manuscript.

## Figures and Tables

**Table 1:**
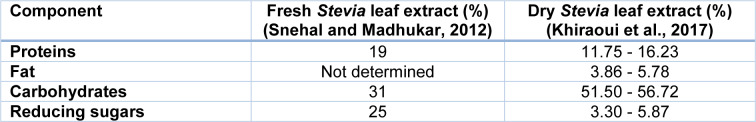
Comparison of biochemical content in fresh and dry extract from *Stevia* leaves

**Table 2:**
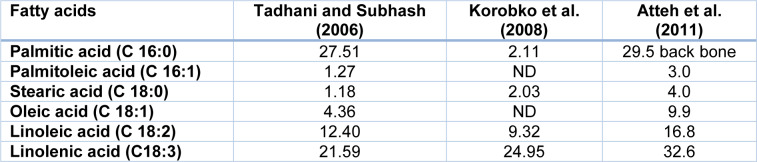
Fatty acid composition of *S. rebaudiana* leaf oil (g/100 g dry mass)

**Table 3:**
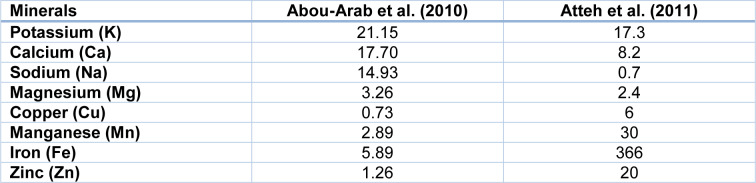
Content of minerals in dried leaves of *S. rebaudiana* (mg/100 g dry mass)

**Table 4:**
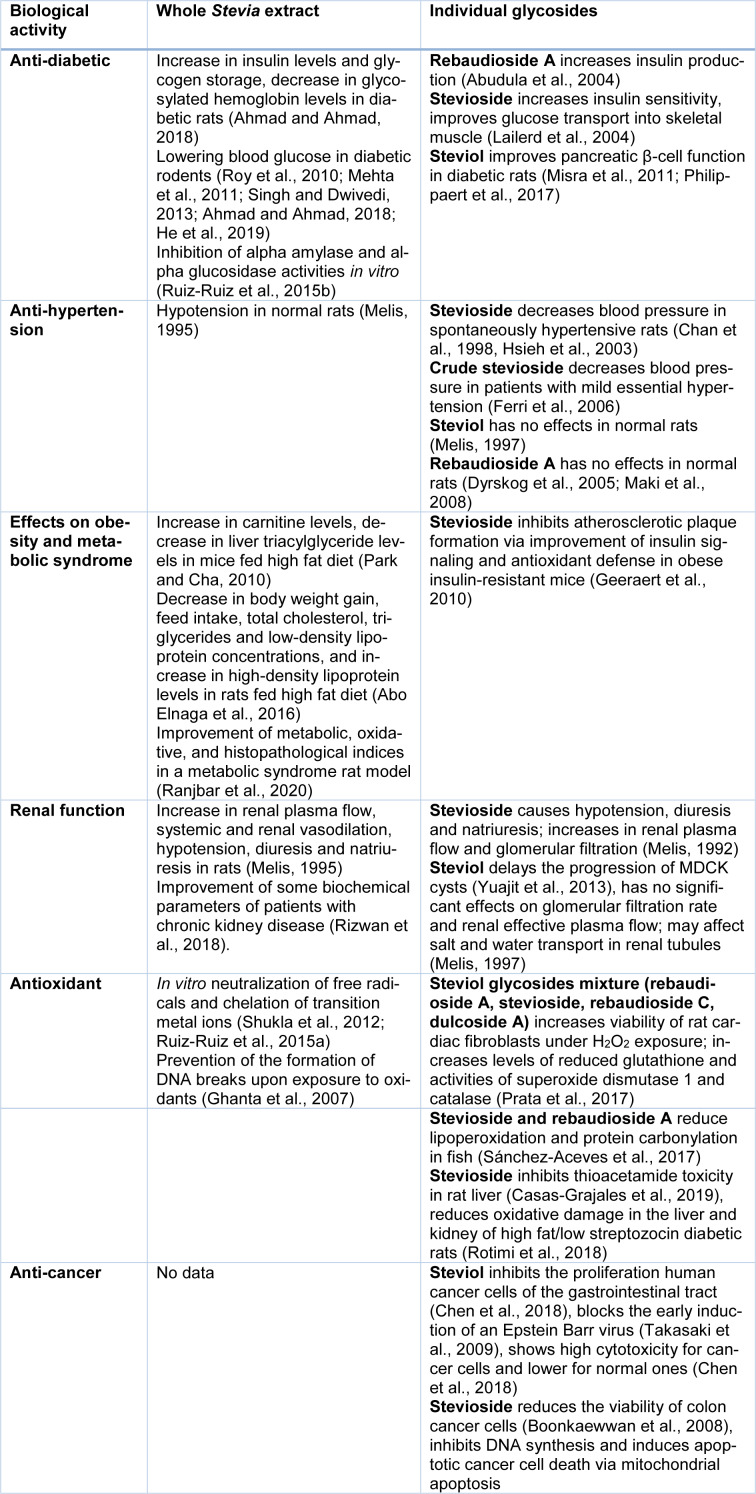
Biological activities of whole *Stevia* leaf extracts and individual *Stevia* glycosides

## References

[1] Abo Elnaga NIE, Massoud MI, Yousef MI, Mohamed HHA (2016). Effect of Stevia sweetener consumption as non-caloric sweetening on body weight gain and biochemical’s parameters in overweight female rats. Ann Agric Sci.

[2] Abou-Arab AE, Abou-Arab AA, Abou-Salem FM (2010). Physico-chemical assessment of natural sweeteners steviosides produced from Stevia rebaudiana Bertoni plant. Afric J Food Sci.

[3] Abudula R, Jeppesen PB, Rolfsen SED, Xiao J, Hermansen K (2004). Rebaudioside A potently stimulates insulin secretion from isolated mouse islets: Studies on the dose-, glucose-, and calcium-dependency. Metabolism.

[4] Aguero SD, Onate G, Rivera HP (2014). Consumption of non-nutritive sweeteners and nutritional status in 10-16-year-old students. Arch Argent Pediatr.

[5] Ahmad U, Ahmad RS (2018). Anti-diabetic property of aqueous extract of Stevia rebaudiana Bertoni leaves in Streptozotocin-induced diabetes in albino rats. BMC Complement Altern Med.

[6] Ahmad U, Ahmad RS, Arshad MS, Mushtaq Z, Hussain SM, Hameed A (2018). Antihyperlipidemic efficacy of aqueous extract of Stevia rebaudiana Bertoni in albino rats. Lipids Health Dis.

[7] Ajami M, Seyfi M, Hosseini FAP, Naseri P, Velayati A, Mahmoudnia F (2020). Effects of Stevia on glycemic and lipid profile of type 2 diabetic patients: A randomized controlled trial. Avicenna J Phytomed.

[8] Allen AL, McGeary JE, Hayes JE (2013). Rebaudioside A and rebaudioside D bitterness do not covary with acesulfame-K bitterness or polymorphisms in TAS2R9 and TAS2R31. Chemosens Percept.

[9] Anton SD, Martin CK, Han H, Coulon S, Cefalu WT, Geiselman P (2010). Effects of Stevia, aspartame, and sucrose on food intake, satiety, and postprandial glucose and insulin levels. Appetite.

[10] Arya A, Kumar S, Kasana MS (2012). Anti-inflammatory activity of in vitro regenerated calli and in vivo plant of Stevia rebaudiana (Bert.) Bertoni. J Sci Ind Res.

[11] Ashwell M (2015). Stevia, nature’s zero-calorie sustainable sweetener. Nutr Today.

[12] Atteh J, Onagbesan O, Tona K, Buyse J, Decuypere E, Geuns J (2011). Potential use of Stevia rebaudiana in animal feeds. Arch de Zootec.

[13] Barriocanal LA, Palacios M, Benitez G, Benitez S, Jimenez JT, Jimenez N (2008). Apparent lack of pharmacological effect of steviol glycosides used as sweeteners in humans. A pilot study of repeated exposures in some normotensive and hypotensive individuals and in Type 1 and Type 2 diabetics. Regul Toxicol Pharmacol.

[14] Bayliak MM, Abrat OB, Storey JM, Storey KB, Lushchak VI (2019). Interplay between diet-induced obesity and oxidative stress: Comparison between Drosophila and mammals. Comp Biochem Physiol A.

[15] Boonkaewwan C, Ao M, Toskulkao C, Rao MC (2008). Specific immunomodulatory and secretory activities of stevioside and steviol in intestinal cells. J Agric Food Chem.

[16] Boonkaewwan C, Burodom A (2013). Anti-inflammatory and immunomodulatory activities of stevioside and steviol on colonic epithelial cells. J Sci Food Agric.

[17] Brandle JE, Telmer PG (2007). Steviol glycoside biosynthesis. Phytochemistry.

[18] Carakostas MC, Curry LL, Boileau AC, Brusick DJ (2008). Overview: The history, technical function and safety of rebaudioside A, a naturally occurring steviol glycoside, for use in food and beverages. Food Chem Toxicol.

[19] Carrera-Lanestosa A, Moguel-Ordóñez Y, Segura-Campos M (2017). Stevia rebaudiana Bertoni: A natural alternative for treating diseases associated with metabolic syndrome. J Med Food.

[20] Casas-Grajales S, Ramos-Tovar E, Chávez-Estrada E, Alvarez-Suarez D, Hernández-Aquino E, Reyes-Gordillo K (2019). Antioxidant and immunomodulatory activity induced by stevioside in liver damage: In vivo, in vitro and in silico assays. Life Sci.

[21] Ceunen S, Wim DB, Compernolle F, Mai AH, Geuns JMC (2013). Diterpene glycosides from Stevia phlebophylla A. Gray. Carbohydr Res.

[22] Chan P, Xu DY, Liu JC, Chen YJ, Tomlinson B, Huang WP (1998). The effect of stevioside on blood pressure and plasma catecholamines in spontaneously hypertensive rats. Life Sci.

[23] Chand G, Kumar S (eds) (2016). Crop diseases and their management. Integrated approaches.

[24] Chen J, Xia Y, Sui X, Peng Q, Zhang T, Li J (2018). Steviol, a natural product inhibits proliferation of the gastrointestinal cancer cells intensively. Oncotarget.

[25] Cho BO, Ryu HW, So Y, Cho JK, Woo HS, Jin CH (2013). Anti-inflammatory effect of austroinulin and 6-O-acetyl-austroinulin from Stevia rebaudiana in lipopolysaccharide-stimulated RAW264.7 macrophages. Food Chem Toxicol.

[26] Contreras S (2013). Anticariogenic properties and effects on periodontal structures of Stevia rebaudiana Bertoni. Narrative review. J Oral Res.

[27] Das S, Das AK, Murphy RA, Punwani IC, Nasution MP, Kinghorn AD (1992). Evaluation of the cariogenic potential of the Intense natural sweeteners stevioside and rebaudioside A. Caries Res.

[28] De Slavutzky SMB (2010). Stevia and sucrose effect on plaque formation. J Verbrauch Lebensm.

[29] Dyrskog SE, Jeppesen PB, Chen J, Christensen LP, Hermansen K (2005). The diterpene glycoside, rebaudioside A, does not improve glycemic control or affect blood pressure after eight weeks treatment in the Goto-Kakizaki rat. Rev Diabet Stud.

[30] Farhat G, Berset V, Moore L (2019). Effects of stevia extract on postprandial glucose response, satiety and energy intake: A three-arm crossover trial. Nutrients.

[31] Ferlay J, Colombet M, Soerjomataram I, Dyba T, Randi G, Bettio M (2018). Cancer incidence and mortality patterns in Europe: Estimates for 40 countries and 25 major cancers in 2018. Eur J Cancer.

[32] Ferri LAF, Alves-Do-Prado W, Yamada SS, Gazola S, Batista MR, Bazotte RB (2006). Investigation of the antihypertensive effect of oral crude stevioside in patients with mild essential hypertension. Phytother Res.

[33] Gamboa F, Chaves M (2012). Antimicrobial potential of extracts from Stevia rebaudiana leaves against bacteria of importance in dental caries. Acta Odontol Latinoam.

[34] Garaschuk O, Semchyshyn HM, Lushchak VI (2018). Healthy brain aging: Interplay between reactive species, inflammation and energy supply. Ageing Res Rev.

[35] Gaweł-Bęben K, Bujak T, Nizioł-Łukaszewska Z, Antosiewicz B, Jakubczyk A, Karaś M (2015). Stevia rebaudiana Bert. leaf wxtracts as a multifunctional source of natural antioxidants. Molecules.

[36] Geeraert B, Crombé F, Hulsmans M, Benhabilès N, Geuns JM, Holvoet P (2010). Stevioside inhibits atherosclerosis by improving insulin signaling and antioxidant defense in obese insulin-resistant mice. Int J Obes (Lond.

[37] Ghanta S, Banerjee A, Poddar A, Chattopadhyay S (2007). Oxidative DNA damage preventive activity and antioxidant potential of Stevia rebaudiana (Bertoni) Bertoni, a natural sweetener. J Agric Food Chem.

[38] Giacaman RA, Campos P, Muñoz-Sandoval C, Castro RJ (2013). Cariogenic potential of commercial sweeteners in an experimental biofilm caries model on enamel. Arch Oral Biol.

[39] Goyal SK, Samsher, Goyal RK (2010). Stevia a bio-sweetener: a review. Inter J Food Sci Nutr.

[40] He J, Zhu NL, Kong J, Peng P, Li LF, Wei XL (2019). A newly discovered phenylethanoid glycoside from Stevia rebaudiana Bertoni affects insulin secretion in rat INS-1 islet β cells. Molecules.

[41] Heyden T How did Stevia get mainstream? BBC News Magazine, 4 June 2013. https://www.bbc.com/news/magazine-22758059.

[42] Hsieh MH, Chan P, Sue YM, Liu JC, Liang TH, Huang TY (2003). Efficacy and tolerability of oral stevioside in patients with mild essential hypertension: A two-year, randomized, placebo-controlled study. Clin Ther.

[43] Ibrahim MA, Rodenburg DL, Alves K, Perera WH, Fronczek FR, Bowling J (2016). Rebaudiosides R and S, minor diterpene glycosides from the leaves of Stevia rebaudiana. J Nat Prod.

[44] Jentzsch P, Torrico-Vallejos S, Mendieta-Brito S, Ramos LA, Ciobotă V (2016). Detection of counterfeit Stevia products using a handheld Raman spectrometer. Vib Spectrosc.

[45] Jeong Y, Lee HJ, Jin GH, Park YD, Choi DS, Kang MA (2010). Anti-inflammatory activity of Stevia rebaudiana in LPS-induced RAW 264.7 cells. J Food Sci Nutr.

[46] Jeppesen PB, Gregersen S, Poulsen CR, Hermansen K (2000). Stevioside acts directly on pancreatic β cells to secrete insulin: Actions independent of cyclic adenosine monophosphate and adenosine triphosphate-sensitivie K+-channel activity. Metabolism.

[47] Kaplan B, Turgut K (2019). Improvement of rebaudioside A diterpene glycoside content in Stevia rebaudiana Bertoni using clone selection. Turk J Agric Forestry.

[48] Khiraoui A, Bakha M, Amchra F, Ourouadi S, Abdelali B, Al-Faiz C (2017). Nutritional and biochemical properties of natural sweeteners of six cultivars of Stevia rebaudiana Bertoni leaves grown in Morocco. J Environ Sci (China).

[49] Kim IS, Yang M, Lee OH, Kang SN (2011). The antioxidant activity and the bioactive compound content of Stevia rebaudiana water extracts. LWT Food Sci Technol.

[50] Konoshima T, Takasaki M (2002). Cancer-chemopreventive effects of natural sweeteners and related compounds. Pure Appl Chem.

[51] Korobko NV, Turko YA, Shokun VV, Chernyak EN, Pokrovskii LM, Smetankina ON (2008). GC-MS investigations. II. Lipid composition of Stevia rebaudiana. Chem Nat Compd.

[52] Lailerd N, Saengsirisuwan V, Sloniger JA, Toskulkao C, Henriksen EJ (2004). Effects of stevioside on glucose transport activity in insulin-sensitive and insulin-resistant rat skeletal muscle. Metabolism.

[53] Lohner S, Toews I, Meerpohl JJ (2017). Health outcomes of non-nutritive sweeteners: Analysis of the research landscape. J Nutr.

[54] Ma MS, Blanksma NG (2015). Stevia in the fight against dental caries. Ned Tijdschr Tandheelkd.

[55] Maki KC, Curry LL, Reeves MS, Toth PD, McKenney JM, Farmer MV (2008). Chronic consumption of rebaudioside A, a steviol glycoside, in men and women with type 2 diabetes mellitus. Food Chem Toxicol.

[56] Marcinek K, Krejpcio Z (2015). Stevia rebaudiana Bertoni – chemical composition and functional properties. Acta Sci Pol Technol Aliment.

[57] Mazzei-Planas G, Kuc J (1968). Contraceptive properties of Stevia rebaudiana. Science.

[58] Mehta B, Jain D, Misra H, Soni M, Silawat N, Mehta D (2011). Antidiabetic activity of medium-polar extract from the leaves of Stevia rebaudiana Bert. (Bertoni) on alloxan-induced diabetic rats. J Pharm Bioallied Sci.

[59] Melis MS (1995). Chronic administration of aqueous extract of Stevia rebaudiana in rats: renal effects. J Ethnopharmacol.

[60] Melis MS (1999). Effects of chronic administration of Stevia rebaudiana on fertility in rats. J Ethnopharmacol.

[61] Melis MS (1997). Effects of steviol on renal function and mean arterial pressure in rats. Phytomedicine.

[62] Melis MS (1992). Stevioside effect on renal function of normal and hypertensive rats. J Ethnopharmacol.

[63] Michels B, Zwaka H, Bartels R, Lushchak O, Franke K, Endres T (2018). Memory enhancement by ferulic acid ester across species. Sci Adv.

[64] Midmore D, Rank AH (2002). A New rural industry - Stevia - to replace imported chemical sweeteners. Report for the Rural Industries Research and Development Corporation.

[65] Milani PG, Formigoni M, Lima YC, Piovan S, Peixoto GML, Camparsi DM (2017). Fortification of the whey protein isolate antioxidant and antidiabetic activity with fraction rich in phenolic compounds obtained from Stevia rebaudiana (Bert.). Int J Food Sci Nutr.

[66] Misra H, Soni M, Silawat N, Mehta D, Mehta BK, Jain DC (2011). Antidiabetic activity of medium-polar extract from the leaves of Stevia rebaudiana Bert. (Bertoni) on alloxan-induced diabetic rats. J Pharm Bioallied Sci.

[67] Mizushina Y, Akihisa T, Ukiya M, Hamasaki Y, Murakami-Nakai C, Kuriyama I (2005). Structural analysis of isosteviol and related compounds as DNA polymerase and DNA topoisomerase inhibitors. Life Sci.

[68] Neuwirth ET (2020). Why you should sweeten with Reb M. Bayn Europe. https://www.bayneurope.com/en/why-you-should-sweeten-with-reb-m/182897.

[69] Nikiforov AI, Rihner MO, Eapen AK, Thomas JA (2013). Metabolism and toxicity studies supporting the safety of rebaudioside D. Int J Toxicol.

[70] Ohtani K, Aikawa Y, Kasai R, Chou W-H, Yamasaki K, Tanaka O (1992). Minor diterpene glycosides from sweet leaves of Rubus suavissimus. Phytochemistry.

[71] Olsson K, Carlsen S, Semmler A, Simón E, Mikkelsen MD, Møller BL (2016). Microbial production of next-generation Stevia sweeteners. Microb Cell Factories.

[72] Onopiuk A, Tokarzewicz A, Gorodkiewicz E (2015). Cystatin C: a kidney function biomarker. Adv Clin Chem.

[73] Oparil S, Acelajado MC, Bakris GL, Berlowitz DR, Cífková R, Dominiczak AF (2018). Hypertension. Nat Rev Dis Primers.

[74] Park JE, Cha YS (2010). Stevia rebaudiana Bertoni extract supplementation improves lipid and carnitine profiles in C57BL/6J mice fed a high-fat diet. J Sci Food Agric.

[75] Paul S, Sengupta S, Bandyopadhyay TK, Bhattacharyya A (2012). Stevioside induced ROS-mediated apoptosis through mitochondrial pathway in human breast cancer cell line MCF-7. Nutr Cancer.

[76] Perera WH, Ghiviriga I, Rodenburg DL, Alves K, Bowling JJ, Avula B (2017). Rebaudiosides T and U, minor C-19 xylopyranosyl and arabinopyranosyl steviol glycoside derivatives from Stevia rebaudiana (Bertoni) Bertoni. Phytochemistry.

[77] Periche A, Castelló ML, Heredia A, Escriche I (2015). Influence of drying method on steviol glycosides and antioxidants in Stevia rebaudiana leaves. Food Chem.

[78] Pezzuto JM, Compadre CM, Swanson SM, Nanayakkara D, Kinghorn AD (1985). Metabolically activated steviol, the aglycone of stevioside, is mutagenic. Proc Natl Acad Sci.

[79] Philippaert K, Pironet A, Mesuere M, Sones W, Vermeiren L, Kerselaers S (2017). Steviol glycosides enhance pancreatic beta-cell function and taste sensation by potentiation of TRPM5 channel activity. Nat Commun.

[80] Piskovatska V, Strilbytska O, Koliada A, Vaiserman A, Lushchak O, Harris JR, Korolchuk VI (2019). Health benefits of anti-aging drugs. Biochemistry and cell biology of ageing: Part II clinical science.

[81] Prakash I, Markosyan A, Bunders C (2014). Development of next generation Stevia sweetener: Rebaudioside M. Foods.

[82] Prata C, Zambonin L, Rizzo B, Maraldi T, Angeloni C, Vieceli Dalla Sega F (2017). Glycosides from Stevia rebaudiana Bertoni possess insulin-mimetic and antioxidant activities in rat cardiac fibroblasts. Oxid Med Cell Longev.

[83] Preethi D, Sridhar TM, Josthna P, Naidu CV (2011). Studies on antibacterial activity, phytochemical analysis of Stevia rebaudiana (Bert.). An important calorie free biosweetner. J Ecobiotech.

[84] Purkayastha S, Kwok D (2020). Metabolic fate in adult and pediatric population of steviol glycosides produced from Stevia leaf extract by different production technologies. Regul Toxicol Pharmacol.

[85] Ramesh K, Singh V, Megeji NW (2006). Cultivation of Stevia [Stevia rebaudiana (Bert.) Bertoni]: a comprehensive review. Adv Agron.

[86] Ranjbar T, Nekooeian AA, Tanideh N, Koohi‐Hosseinabadi O, Masoumi SJ, Amanat S (2020). A comparison of the effects of Stevia extract and metformin on metabolic syndrome indices in rats fed with a high‐fat, high‐sucrose diet. J Food Biochem.

[87] Reidy K, Kang HM, Hostetter T, Susztak K (2014). Molecular mechanisms of diabetic kidney disease. J Clin Investig.

[88] Rizwan F, Rashid HU, Yesmine S, Monjur F, Chatterjee TK (2018). Preliminary analysis of the effect of Stevia (Stevia rebaudiana) in patients with chronic kidney disease (stage I to stage III). Contemp Clin Trials Commun.

[89] Rotimi SO, Rotimi OA, Adelani IB, Onuzulu C, Obi P, Okungbaye R (2018). Stevioside modulates oxidative damage in the liver and kidney of high fat/low streptozocin diabetic rats. Heliyon.

[90] Roy B, Kujur R, Singh V, Ram M, Yadava H, Singh K (2010). Antidiabetic activity and phytochemical screening of crude extract of Stevia rebaudiana in alloxan-induced diabetic rats. Pharmacogn Res.

[91] Ruiz-Ruiz JC, Moguel-Ordoñez YB, Matus-Basto AJ, Segura-Campos MR (2015). Antidiabetic and antioxidant activity of Stevia rebaudiana extracts (Var. Morita) and their incorporation into a potential functional bread. J Food Sci Technol.

[92] Ruiz-Ruiz JC, Moguel-Ordoñez YB, Matus-Basto AJ, Segura-Campos MR (2015). Antioxidant capacity of leaf extracts from two Stevia rebaudiana Bertoni varieties adapted to cultivation in Mexico. Nutr Hosp.

[93] Sanches Lopes SM, Francisco MG, Higashi B, de Almeida RTR, Krausová G, Pilau EJ (2016). Chemical characterization and prebiotic activity of fructo-oligosaccharides from Stevia rebaudiana (Bertoni) roots and in vitro adventitious root cultures. Carbohydr Polym.

[94] Sánchez-Aceves LM, Dublán-García O, López-Martínez LX, Novoa-Luna KA, Islas-Flores H, Galar-Martínez M (2017). Reduction of the oxidative stress status using steviol glycosides in a fish model (Cyprinus carpio). BioMed Res Int.

[95] Savita SM, Sheela K, Sunanda S, Shankar AG, Ramakrishna P (2004). Stevia rebaudiana – a functional component for food industry. Hum Ecol.

[96] Sehar I, Kaul A, Bani S, Pal HC, Saxena AK (2008). Immune up regulatory response of a non-caloric natural sweetener, stevioside. Chem Biol Interact.

[97] Shibata H, Sawa Y, Oka T, Sonoke S, Kim KK, Yoshioka M (1995). Steviol and steviol-glycoside: glucosyltransferase activities in Stevia rebaudiana Bertoni - purification and partial characterization. Arch Biochem Biophys.

[98] Shukla S, Mehta A, Mehta P, Bajpai VK (2012). Antioxidant ability and total phenolic content of aqueous leaf extract of Stevia rebaudiana Bert. Exp Toxicol Pathol.

[99] Singh P, Dwivedi P (2013). Two-stage culture procedure using thidiazuron for efficient micropropagation of Stevia rebaudiana, an anti-diabetic medicinal herb. 3 Biotech.

[100] Singh SD, Rao GP (2005). Stevia: The herbal sugar of 21st century. Sugar Tech.

[101] Snehal P, Madhukar K (2012). Quantitative estimation of biochemical content of various extracts of Stevia rebaudiana leaves. Asian J Pharm Clin Res.

[102] Starratt AN, Kirby CW, Pocs R, Brandle JE (2002). Rebaudioside F, a diterpene glycoside from Stevia rebaudiana. Phytochemistry.

[103] Tadhani MB, Patel VH, Subhash R (2007). In vitro antioxidant activities of Stevia rebaudiana leaves and callus. J Food Compos Anal.

[104] Tadhani MB, Subhash R (2006). Preliminary studies on Stevia rebaudiana leaves: proximal composition, mineral analysis and phytochemical screening. Int J Med Sci.

[105] Takasaki M, Konoshima T, Kozuka M, Tokuda H, Takayasu J, Nishino H (2009). Cancer preventive agents. Part 8: Chemopreventive effects of stevioside and related compounds. Bioorg Med Chem.

[106] Tao Z, Shi A, Zhao J (2015). Epidemiological perspectives of diabetes. Cell Biochem Biophys.

[107] Uçar A, Yılmaz S, Yılmaz Ş, Kılıç MS (2017). A research on the genotoxicity of Stevia in human lymphocytes. Drug Chem Toxicol.

[108] Uhler B, Yang Z (2018). Rebaudioside A and other unreported steviol glycoside isomers found in the sweet tea (Rubus suavissimis) leaf. Phytochem Lett.

[109] Urban JD, Carakostas MC, Taylor SL (2015). Steviol glycoside safety: Are highly purified steviol glycoside sweeteners food allergens?. Food Chem Toxicol.

[110] Vaiserman A, Koliada A, Lushchak O (2020). Neuroinflammation in pathogenesis of Alzheimer's disease: Phytochemicals as potential therapeutics. Mech Ageing Dev.

[111] Vaiserman A, Koliada A, Zayachkivska A, Lushchak O (2020). Curcumin: A therapeutic potential in ageing-related disorders. PharmaNutrition.

[112] Vaiserman A, Lushchak O (2019). Developmental origins of type 2 diabetes: Focus on epigenetics. Ageing Res Rev.

[113] Vaiserman A, Lushchak O (2019). Prenatal malnutrition-induced epigenetic dysregulation as a risk factor for type 2 diabetes. Int J Genomics.

[114] Vallon V, Komers R (2011). Pathophysiology of the diabetic kidney. Compr Physiol.

[115] Vandana K, Reddy VC, Sudhir KM, Kumar K, Raju SH, Babu lN (2017). Effectiveness of Stevia as a mouthrinse among 12–15-year-old schoolchildren in Nellore district, Andhra Pradesh - A randomized controlled trial. J Indian Soc Periodontol.

[116] Wang M, Li H, Xu F, Gao X, Li J, Xu S (2018). Diterpenoid lead stevioside and its hydrolysis products steviol and isosteviol: Biological activity and structural modification. Eur J Med Chem.

[117] Yasukawa K, Kitanaka S, Seo S (2002). Inhibitory effect of stevioside on tumor promotion by 12-O-tetradecanoylphorbol-13-acetate in two-stage carcinogenesis in mouse Skin. Biol Pharm Bull.

[118] Yuajit C, Homvisasevongsa S, Chatsudthipong L, Soodvilai S, Muanprasat C, Chatsudthipong V (2013). Steviol reduces MDCK cyst formation and growth by inhibiting CFTR channel activity and promoting proteasome-mediated CFTR degradation. PLoS One.

[119] Zaman N, Crosson M, Whitehurst T, Barrette E, Henderson J, Rajchel D (2015). Llewellyn’s 2015 herbal almanac: herbs for growing and gathering, cooking and crafts, health and beauty, history, myth and lore.

[120] Zimmet P, Alberti KG, Magliano DJ, Bennett PH (2016). Diabetes mellitus statistics on prevalence and mortality: facts and fallacies. Nat Rev Endocrinol.

